# Prenatal manifestations and perinatal outcomes in congenital myotonic dystrophy: clinical patterns and diagnostic implications

**DOI:** 10.1515/crpm-2026-0013

**Published:** 2026-08-10

**Authors:** Nawras Zayat, Tori Aspir, Eliane Shinder, Susan Wu, Emily Suskin, Sameer Khan, Sara Rabin-Havt, Pe’er Dar, Georgios Doulaveris

**Affiliations:** Department of Obstetrics and Gynecology, Division of Maternal-Fetal Medicine, Montefiore Medical Center, Albert Einstein College of Medicine, Bronx, New York, NY, USA; Department of Obstetrics and Gynecology and Women’s Health, Montefiore Medical Center, Albert Einstein College of Medicine, Bronx, New York, NY, USA; Department of Obstetrics and Gynecology, Stony Brook University Hospital, Stony Brook University, Renaissance School of Medicine, Stony Brook, New York, NY, USA; Department of Obstetrics and Gynecology, Division of Medical and Reproductive Genetics, Montefiore Medical Center, Albert Einstein College of Medicine, Bronx, New York, NY, USA

**Keywords:** polyhydramnios, congenital myotonic dystrophy, pregnancy, pregnancy complications, hydrops fetalis, grip myotonia

## Abstract

**Objectives:**

This work aims to characterize the clinical manifestations and diagnostic challenges associated with pregnancies affected by congenital myotonic dystrophy through a detailed case report of an individual seen in our center and a comprehensive case series overview.

**Case presentation:**

A 33-year-old woman presented at 33 weeks-gestation with symptomatic severe polyhydramnios (AFI 55.2) and an otherwise uncomplicated prenatal course with no anomalies on ultrasound. She underwent amnioreduction, which initially revealed normal genetic testing results (46,XX karyotype and normal microarray). At 34 weeks, she underwent repeat cesarean delivery for new-onset non-immune hydrops fetalis. Because of the hydrops, severe hypotonia, and respiratory distress requiring intubation of the neonate, further genetic testing was performed and was positive for congenital myotonic dystrophy (1,880 CTG repeats in DMPK). This testing also indicated that the mother had >200 repeats, consistent with myotonic dystrophy type 1.

**Conclusions:**

Idiopathic polyhydramnios and non-immune hydrops fetalis, even in the absence of structural anomalies, should prompt consideration of neuromuscular conditions such as congenital myotonic dystrophy in the differential diagnosis.

## Introduction

Myotonic dystrophy type 1 (DM1) is the most common inherited muscular dystrophy in adults, with an estimated prevalence of 1 in 8,000 individuals worldwide [1]. It is an autosomal dominant disorder caused by an expanded CTG trinucleotide repeat in the *DMPK* gene on chromosome 19q13.3 [2]. The clinical spectrum is broad and includes adult-onset, childhood-onset, and congenital forms, with severity generally correlating with repeat size.

Congenital myotonic dystrophy (CDM) represents the most severe phenotype, typically defined by neonatal onset of hypotonia, respiratory insufficiency, and feeding difficulties, and is associated with high neonatal morbidity and mortality [3]. Affected infants often require prolonged ventilatory support, and mortality rates in the neonatal period have been reported to be 30–40 % [3]. Nearly all cases of CDM are maternally inherited due to genetic anticipation, with repeat expansions frequently exceeding 1,000 [2].

In the absence of a known family history, prenatal identification of CDM remains difficult. Unlike structural anomalies that can be readily identified on ultrasound, the most common prenatal features of CDM are nonspecific and include polyhydramnios, reduced or absent fetal movement, and talipes or clubfoot [4]. Polyhydramnios is thought to result from impaired fetal swallowing secondary to neuromuscular dysfunction, while hydrops fetalis, though less common, has also been reported in association with CDM [5]. These findings overlap with other etiologies, potentially making CDM an underrecognized cause of idiopathic polyhydramnios and unexplained non-immune hydrops.

Recognition of these associations has important implications for prenatal counseling, genetic testing, and delivery planning. In this report, we present a case of severe polyhydramnios progressing to hydrops fetalis in the third trimester, ultimately diagnosed as CDM following neonatal genetic testing. We also review the literature on CDM in pregnancy to underscore the diagnostic challenges and to highlight the importance of including neuromuscular disorders in the differential diagnosis of polyhydramnios and non-immune hydrops fetalis.

## Case presentation

A 33-year-old pregnant woman, gravida 2, para 1, presented at 33 weeks’ gestation to a Maternal-Fetal Medicine outpatient clinic at a tertiary care center for consultation for polyhydramnios in the third trimester. The pregnancy was otherwise uncomplicated; first and second trimester anatomy ultrasounds were unremarkable with no fetal anomalies identified. Maternal cytomegalovirus and toxoplasmosis serologies were negative.

Initial evaluation revealed marked polyhydramnios (AFI 32.5 cm). The differential diagnosis included unrecognized maternal diabetes mellitus, fetal gastrointestinal anomaly (e.g., bowel obstruction, tracheoesophageal fistula), and fetal brain abnormalities associated with impaired swallowing, and neuromuscular disorders. A plan was made for weekly fetal assessment and amnioreduction as required for maternal symptoms.

Three days later, at 33 weeks 5 days’ gestation, the patient presented with abdominal distention, discomfort, and orthopnea. She was found to have severe polyhydramnios with an AFI 55.2 cm, and symptoms were attributed to impaired diaphragmatic excursion from uterine overdistention. She underwent uncomplicated amnioreduction, during which 4,000 mL of clear amniotic fluid was aspirated and resulted in improved maternal symptoms. Genetic testing on aspirated fluid revealed a normal 46,XX karyotype and a normal chromosomal microarray.

At 34 weeks 6 days, new-onset fetal scalp edema was suspected on serial ultrasonography. She was admitted to the Antepartum service for further evaluation. Subsequent detailed ultrasound confirmed hydrops fetalis with scalp edema and bilateral pleural effusions. Maternal blood type was O Rh-positive with a negative antibody screen. Middle cerebral artery (MCA) Doppler demonstrated a peak systolic velocity (PSV) of 1.26 MoM, within normal range for gestational age. For newly diagnosed non-immune hydrops fetalis, she received antenatal betamethasone and underwent elective repeat cesarean delivery via Pfannenstiel incision.

The neonate was a 2,560 g female with minimal respiratory efforts, cyanosis, and generalized hypotonia. Apgar scores were 2, 6, and 7 at 1, 5, and 10 min, respectively. Examination revealed severe scalp edema (head circumference 36.5 cm, >99th percentile) and generalized hydrops involving multiple fluid compartments. The neonate was intubated in the delivery room and was transferred to the NICU in critical condition.

NICU evaluation noted dysmorphic features (including abnormal ear formation) and diffuse hypotonia. The infant required prolonged invasive ventilation, bilateral chest tubes for pleural effusions, and treatment for persistent hypotension. The course was complicated by chylothorax confirmed with lymphangiogram. Workup for hydrops included a negative infectious evaluation. Pediatric Infectious Disease, Genetics, and Neurology were consulted. Genomic sequencing and early onset myotonia panel confirmed congenital myotonic dystrophy type 1 with >1,880 CTG repeats in the *DMPK* gene on day of life 31.

As the genome sequencing was performed in the mother-baby dyad, the results indicated that it was maternally inherited and the mother had >200 CTG repeats in *DMPK*, consistent with DM1. She denied family history of neuromuscular disease but endorsed nonspecific chronic fatigue and difficulty with fine motor tasks, suggestive of possible grip myotonia. She was referred to Neurology for baseline evaluation and long-term care.

The patient and infant ultimately transferred care to an out-of-state tertiary center for specialized neonatal management. At six-month postpartum follow-up by phone, the mother reported that the infant had undergone percutaneous coronary intervention with stent placement and was successfully extubated. At the time of writing, the infant remains hospitalized on nasal cannula respiratory support and is receiving enteral tube feeds with plans to trial oral feeding. The mother has not yet pursued Cardiology or Neurology follow-up. Records following transfer were not available for independent review.

## Methods

A comprehensive analysis of 27 published cases of pregnancies affected by myotonic dystrophy was conducted, alongside a detailed case report of the patient managed at our center. To gather the case reports of pregnancies affected by myotonic dystrophy, a systematic method was applied to search multiple electronic databases, including MEDLINE, Embase, PubMed, and Google Scholar. Keywords including “myotonic dystrophy”, “congenital myotonic dystrophy”, “pregnancy”, “polyhydramnios”, “case reports” and “fetal hydrops” were used to focus the search. Filters were applied for English-language reports and publication dates through September 2025. Case reports were screened using predefined inclusion criteria: pregnancies that included information on maternal and antepartum management with either prenatal or postnatal diagnosis of myotonic dystrophy. Reports describing only adult-onset DM1 were excluded. Data extraction focused on patient demographics, genetic testing results, pregnancy and delivery complications, and neonatal outcomes. Maternal and neonatal data were then analyzed descriptively. Written informed consent for publication was obtained from the patient. This single-patient case report with literature review was exempt from Institutional Review Board review according to institutional policy. The study was conducted in accordance with the principles of the Declaration of Helsinki.

## Results

### Maternal characteristics

A total of 27 pregnancies were analyzed (Table 1). The included cases were derived from published case reports and case series describing prenatal manifestations and perinatal outcomes of congenital myotonic dystrophy [4], [6], [7], [8], [9], [10]. The mean maternal age was 30.2 ± 5.8 years (range 20–40), with a median of 31 years. Nearly half of mothers were nulliparous (48.1 %), and 40.7 % were multiparous. Parity was not reported in 3/27 (11.1 %). Twenty-five women (92.6 %) were confirmed to have myotonic dystrophy type 1 (DM1). Seven of these women (25.9 %) were diagnosed prior to pregnancy, while for the other 16 cases (59.3 %), the maternal diagnosis was made only after the affected neonate was identified. A known or suspected family history of DM1 was reported in 25.9 % of cases. Grip myotonia was documented in 13 mothers (48.1 %), while it was absent in 1 (3.7 %) and not reported in the remainder.

**Table 1:** Maternal characteristics of pregnancies affected by myotonic dystrophy (n=27).

Characteristic	n (%)
Parity	Nulliparous 13 (48.2 %); multiparous 11 (40.7 %); not reported 3 (11.1 %)
DM1 diagnosed before pregnancy	7 (25.9 %)
DM1 diagnosed after neonatal diagnosis	16 (59.3 %)
Family history of DM1	7 (25.9 %)
Maternal grip myotonia present	13 (48.1 %)
Maternal grip myotonia absent	1 (3.7 %)

### Prenatal findings

Polyhydramnios was present in 22 cases (81.5 %), diagnosed at a mean gestational age of 30.5 ± 3.7 weeks (Table 2). The mean amniotic fluid index (AFI) or deepest vertical pocket (DVP) at diagnosis was 36.1 ± 7.7 cm. When quantitative measurements were available, most cases met criteria for moderate-to-severe polyhydramnios. However, severity could not be uniformly classified across all reports because AFI or DVP measurements were not consistently provided. Trimester of diagnosis was specified in 15 cases: 4 in the second trimester and 11 in the third. Amnioreduction was planned or performed in 4 cases (18.2 %), with a recorded range of 4,000–9,300 mL removed (n=2). Decreased fetal movement was noted in 29.6 % of pregnancies. Additional ultrasound anomalies were frequent and included ventriculomegaly (25.9 %), clubfoot/talipes (18.5 %), hydrops fetalis (11.1 %), pleural effusion (14.8 %, of which 3/4 were associated with hydrops), hydronephrosis (7.4 %), pulmonary hypoplasia (7.4 %), diaphragmatic defects (7.4 %), absent cavum septi pellucidi (3.7 %), and preeclampsia with severe features (3.7 %) [8], [9], [10], [11]. In 4 pregnancies (14.8 %), polyhydramnios was the only reported abnormality. Pulmonary hypoplasia was reported based on prenatal imaging findings described in the original case reports, including reduced thoracic dimensions and associated thoracic abnormalities predictive of pulmonary hypoplasia; however, specific diagnostic criteria were not consistently provided (Table 3).

**Table 2:** Prenatal findings, genetic evaluation, and delivery outcomes in pregnancies affected by myotonic dystrophy (n=27).

Finding	n (%)
**Prenatal findings**	
Polyhydramnios	22 (81.5 %)
Polyhydramnios, isolated	4 (14.8 %)
Polyhydramnios with additional findings	18 (66.7 %)
Decreased fetal movement	8 (29.6 %)
Ventriculomegaly	7 (25.9 %)
Clubfoot/talipes	5 (18.5 %)
Hydrops fetalis	3 (11.1 %)
Pleural effusion	4 (14.8 %)
Hydronephrosis	2 (7.4 %)
Pulmonary hypoplasia	2 (7.4 %)
Diaphragmatic defects	2 (7.4 %)
Absent cavum septum pellucidi (CSP)	1 (3.7 %)
Preeclampsia with severe features	1 (3.7 %)
**Genetic evaluation**	
Prenatal DM1 diagnosis	6 (22.2 %)
Prenatal cfDNA screening	3 (11.1 %)
Diagnostic genetic testing in pregnancy	7 (25.9 %)
**Delivery outcomes**	
Preterm delivery	13 (48.2 %)
Full-term delivery	13 (48.2 %)
Vaginal delivery	3 (11.1 %)
Cesarean delivery	22 (81.5 %)
Termination	1 (3.7 %)

**Table 3:** Neonatal outcomes in infants with congenital myotonic dystrophy (n=27).

Finding	n (%)
Birthweight <1,500 g	3 (13.0 %)
Birthweight 1,500–2,500 g	8 (34.8 %)
Birthweight 2,500–4,000 g	11 (47.8 %)
Birthweight >4,000 g	0 (0 %)
Macrocephaly	9 (33.3 %)
Hypotonia	25 (92.6 %)
Mechanical intubation/respiratory support	20 (74.1 %)
Chylothorax	2 (7.4 %)
Intracranial hemorrhage	1 (3.7 %)
Apgar <7 at 1 min	19 (70.4 %)
Apgar <7 at 5 min	15 (55.6 %)
Neonatal mortality	8 (29.6 %)

### Pregnancy and delivery outcomes

Prenatal diagnosis of DM1 was established in 6 cases (22.2 %). Among these, 4 (66.7 %) occurred in pregnancies where the mother had a previously known diagnosis of myotonic dystrophy, while 2 (33.3 %) were identified in mothers without a prior diagnosis. Three pregnancies (11.1 %) underwent cell-free DNA screening, and 7 (25.9 %) underwent invasive diagnostic genetic testing (table 2). Preterm delivery occurred in 13 cases (48.2 %), while 13 pregnancies (48.2 %) delivered at term. One pregnancy was electively terminated (3.7 %). The cesarean delivery rate was 81.5 % (n=22), with 3 vaginal deliveries (11.1 %).

### Neonatal outcomes

Birthweight data were available for 23 neonates: 13.0 % weighed <1,500 g, 34.8 % between 1,500 and 2,500 g, and 47.8 % between 2,500 and 4,000 g; none weighed >4,000 g (table 2). Macrocephaly was observed in 33.3 % of cases. Hypotonia was nearly universal (92.6 %), and 74.1 % required mechanical ventilation or other respiratory support. Chylothorax was reported in 2 neonates (7.4 %), and 1 neonate (3.7 %) experienced intracranial hemorrhage. Low Apgar scores were common, with 70.4 % <7 at 1 min and 55.6 % <7 at 5 min. Neonatal mortality occurred in 8 cases (29.6 %).

## Discussion

Congenital myotonic dystrophy (CDM) represents the most severe end of the myotonic dystrophy type 1 (DM1) spectrum and remains a challenging prenatal diagnosis, particularly in the absence of a known family history [1]. The case presented here exemplifies several characteristic prenatal features of CDM, including severe polyhydramnios, decreased fetal movement, and progression to hydrops fetalis. Despite serial ultrasounds revealing no structural anomalies, the fetus developed late-onset hydrops, ultimately prompting delivery. The prenatal differential diagnosis for polyhydramnios and hydrops is broad, while the postnatal picture revealed valuable phenotypic details. This case reinforces the diagnostic ambiguity surrounding CDM in pregnancy and illustrates how CDM can present primarily as unexplained polyhydramnios or hydrops, often without recognized maternal signs of DM1.

A key feature of CDM is impaired fetal swallowing due to early neuromuscular dysfunction, which accounts for the high prevalence of polyhydramnios in affected pregnancies [4], 12]. In our review, polyhydramnios occurred in 81.5 % of cases, consistent with prior literature describing it as the most common prenatal sign [4]. However, polyhydramnios alone has poor specificity; it is most attributed to gastrointestinal obstruction, maternal diabetes, fetal anemia, or structural abnormalities [12]. Only 22.2 % of pregnancies in our review received a prenatal diagnosis of DM1, underscoring the difficulty of recognizing CDM prospectively based on ultrasound findings alone. Importantly, 14.8 % of reported pregnancies demonstrated no abnormalities other than polyhydramnios, like our patient prior to development of hydrops.

Recent evidence supports the diagnostic value of exome sequencing (ES) in isolated polyhydramnios. In a cohort of 40 cases with normal chromosomal microarray, ES identified pathogenic or likely pathogenic variants in 17.5 % of pregnancies, including both mild and moderate-severe cases [11]. Diagnoses included Bartter syndrome, Noonan syndrome, and one case of myotonic dystrophy type 1 diagnosed postnatally [11]. These findings suggest that comprehensive genomic testing may uncover clinically relevant monogenic etiologies even when polyhydramnios appears isolated. Prenatal diagnosis of these conditions may facilitate pregnancy management options, anticipatory counseling, and delivery planning.

Despite advances in prenatal genetic testing, hereditary screening for DM1 remains challenging because it is a CTG repeat-expansion disorder. Standard karyotype, chromosomal microarray, cell-free DNA screening, and many sequencing-based tests do not reliably detect DMPK repeat expansions unless targeted testing is specifically requested [13]. This limitation may contribute to delayed diagnosis in pregnancies presenting with otherwise unexplained polyhydramnios. Careful assessment of maternal symptoms, family history, and consideration of targeted DMPK testing may facilitate earlier recognition of affected pregnancies, particularly when severe unexplained polyhydramnios, decreased fetal movement, or hydrops is present.

Hydrops fetalis, though less frequently reported, appears to be an especially concerning manifestation of severe phenotype abnormalities, including hypotonia, pleural effusion, respiratory distress, and tented mouth facies [6]. In our review, 11.1 % of fetuses developed hydrops, often including pleural effusions. Our patient developed hydrops which although rare, is a feature noted to be associated with CDM. While fetal swallowing impairment and secondary polyhydramnios may theoretically contribute to hydrops, the underlying mechanism in CDM is not well defined, and these findings may present independently.

The lack of maternal diagnosis remains a major barrier to early detection. Although most mothers in our review (92.6 %) were ultimately diagnosed with DM1, only 7 out of 27 (25.9 %) had a known diagnosis prior to pregnancy. In contrast, the majority of cases (16/27, 59.3 %) were identified only after the affected neonate was diagnosed, prompting maternal genetic evaluation. Many women were asymptomatic or had subtle signs such as grip myotonia or chronic fatigue that went unrecognized until evaluation was triggered by the neonatal presentation. These findings emphasize the importance of considering maternal neuromuscular evaluation when unexplained polyhydramnios or fetal hypotonia arises, particularly if the mother reports even minimal motor symptoms or a family history suggestive of DM1.

Neonatal outcomes in CDM remain guarded, particularly for those delivered preterm or presenting with hydrops. In our review, neonatal mortality occurred in 8 cases (29.6 %), and 74.1 % of neonates required mechanical ventilation or other respiratory support. These findings are consistent with prior reports describing neonatal mortality rates approaching 30–40 % in congenital myotonic dystrophy [3]. The neonatal course of our patient, marked by profound hypotonia, severe respiratory insufficiency requiring mechanical ventilation, and chylothorax, reflects the significant morbidity frequently described in affected neonates. Hypotonia was nearly universal in our series (92.6 %), highlighting the severe neuromuscular impairment that typically defines the neonatal presentation of congenital myotonic dystrophy. Although chylothorax is an uncommon and poorly understood complication, it was reported in a small subset of cases in our review and has been associated with more severe disease phenotypes and prolonged respiratory support in prior studies [14]. Although long-term prognoses vary, early survival is closely linked to the degree of respiratory compromise and access to specialized neonatal care [3]. These findings suggest that the presence of additional fetal abnormalities beyond polyhydramnios, such as hydrops or pleural effusions, may be associated with an increased risk of higher neonatal morbidity.

The findings from this case and literature review highlight several important clinical implications. CDM should be considered in pregnancies complicated by unexplained severe polyhydramnios, particularly when associated with decreased fetal movement or hydrops. Targeted maternal evaluation, including assessment for subtle symptoms of DM1, may facilitate earlier diagnosis and appropriate genetic testing. Early multidisciplinary involvement is important because affected neonates frequently require prolonged respiratory support and intensive neonatal care. Increased awareness of the prenatal manifestations of CDM may improve counseling, delivery planning, neonatal preparedness, and care coordination.
